# Neural signatures of promotion versus prevention goal priming: fMRI evidence for distinct cognitive-motivational systems

**DOI:** 10.1017/pen.2019.13

**Published:** 2020-02-03

**Authors:** Allison M. Detloff, Ahmad R. Hariri, Timothy J. Strauman

**Affiliations:** 1 University of Wisconsin-Madison, Madison, WI, USA; 2 Department of Psychology and Neuroscience, Duke University, Durham, NC, USA

**Keywords:** Regulatory focus, Self-regulation, fMRI, Promotion, Prevention, Personal goals, Individual differences

## Abstract

Regulatory focus theory (RFT) postulates two cognitive-motivational systems for personal goal pursuit: the promotion system, which is associated with ideal goals (an individual’s hopes, dreams, and aspirations), and the prevention system, which is associated with ought goals (an individual’s duties, responsibilities, and obligations). The two systems have been studied extensively in behavioral research with reference to differences between promotion and prevention goal pursuit as well as the consequences of perceived attainment versus nonattainment within each system. However, no study has examined the neural correlates of each combination of goal domain and goal attainment status. We used a rapid masked idiographic goal priming paradigm and functional magnetic resonance imaging to present individually selected promotion and prevention goals, which participants had reported previously that they were close to attaining (“match”) or far from attaining (“mismatch”). Across the four priming conditions, significant activations were observed in bilateral insula (Brodmann area (BA) 13) and visual association cortex (BA 18/19). Promotion priming discriminantly engaged left prefrontal cortex (BA 9), whereas prevention priming discriminantly engaged right prefrontal cortex (BA 8/9). Activation in response to promotion goal priming was also correlated with an individual difference measure of perceived success in promotion goal attainment. Our findings extend the construct validity of RFT by showing that the two systems postulated by RFT, under conditions of both attainment and nonattainment, have shared and distinct neural correlates that interface logically with established network models of self-regulatory cognition.

Goal pursuit is among the most fundamental of human psychological activities (Miller, Galanter, & Pribram, 1960). Much of human behavior can be understood to reflect people’s efforts to pursue desired outcomes and avoid undesired ones (Deci & Ryan, 2000). Different types of goals are associated with different motivational states, means of pursuit, and affective/behavioral consequences. The types of goals people pursue and the ways in which they pursue them have been used to account for a broad spectrum of behavioral differences as well as emotional vulnerabilities such as depression (Strauman, 2017). In order for effective goal pursuit to occur, the individual must have the capacity for self-regulation (Hoyle & Gallagher, 2015), particularly including the processes by which individuals identify and pursue goals and assess their progress toward them (Carver & Scheier, 1990). Effective self-regulation is supported by executive functioning skills such as attention, working memory, response inhibition, and task switching (Hofmann, Schmeichel, & Baddeley, 2012; Hofmann, Schmeichel, Friese, & Baddeley, 2010), which operate together to create the capacity to represent and pursue higher order goals, initiate and sustain adaptive motivational orientations, and respond to goal-relevant feedback (Baumeister & Heatherton, 1996; Carver & Scheier, 1981; Hofmann et al., 2012).

Personal goal pursuit has been studied extensively using regulatory focus theory (RFT; Higgins, 1997, 1998). RFT postulates two cognitive-motivational systems that correspond to distinct self-regulatory orientations for attaining desired end states: *prevention* and *promotion*. A prevention orientation emphasizes duties, obligations, and responsibilities, which in turn are related to fundamental needs for security. Goals are viewed as oughts, and there is a strategic concern with, n lay person’s terms, “keeping bad things from happening.” A promotion orientation emphasizes hopes, accomplishments, and advancement, which in turn are related to fundamental needs for nurturance. Goals are viewed as ideals, and there is a strategic emphasis on “making good things happen.” Regulatory focus is a psychological state that varies both across individuals (when construed as a dispositional variable) and within individuals (on a momentary basis across situations). The promotion and prevention systems are postulated to be functionally distinct from the behavioral activation system (BAS; Depue & Iacono, 1989; Gray, 1990) and behavioral inhibition system (BIS; Fowles, 1988; Gray, 1982). For example, while both promotion/prevention and BIS/BAS can be conceptualized broadly in terms of approach and avoidance (Scholer & Higgins, 2008), RFT emphasizes strategic pursuit of higher order goals whereas BIS/BAS refers primarily to spatiotemporal, evolutionarily determined approach/avoidance behaviors (Depue & Collins, 1999; Strauman & Wilson, 2010).

RFT predicts how perceived successes or failures in goal pursuit can give rise to different emotions. Self-discrepancy theory (SDT; Higgins, 1987), a precursor of RFT, proposed that a perceived discrepancy between one’s actual self and one’s ideal versus ought self, which represent motivationally significant self-guides, creates different emotional responses. Attaining or not attaining a particular self-guide was postulated to represent a specific psychological situation (Lewin, 1951), and self-regulation in reference to ideal and ought self-guides illustrates the motivational and affective distinctions between promotion and prevention goal pursuit. According to SDT, believing that one is attaining an ideal (promotion) goal is associated with emotions such as joy, satisfaction, and happiness, whereas believing that one is not attaining such a goal is associated with sadness, disappointment, and frustration. Likewise, believing that one is attaining an ought (prevention) goal is associated with emotions such as quiescence and calmness, whereas believing that one is not attaining such a goal is associated with anxiety, worry, and agitation.

A number of investigators have sought to identify neural correlates of promotion and prevention (e.g., Cunningham et al., 2005; Packer & Cunningham, 2009; Touryan et al., 2007). The logic for such an investigation reflects the hypothesized development of promotion and prevention orientations as derived from the individual’s socialization history (Scholer & Higgins, 2008). Specifically, strength of orientation to promotion versus prevention is postulated to be determined by experienced patterns of caretaker/child interactions (Higgins, 1998; Manian, Papadakis, Strauman, & Essex, 2006). Therefore, individuals would be expected to vary in the strength of neural responses to cues for promotion versus prevention goal pursuit, just as they have been observed to vary with regard to motivational, affective, and behavioral consequences of promotion or prevention goal activation. The initial studies linking regulatory focus with brain activation drew extensively on the broader literature on frontal asymmetry and positive versus negative affectivity (e.g., Coan & Allen, 2003). Amodio, Shah, Sigelman, Brazy, and Harmon-Jones (2004) examined the associations between individual differences in chronic regulatory focus and an EEG measure of resting prefrontal cortex (PFC) activation asymmetry. Individuals with a chronic promotion focus manifested higher levels of resting left PFC activity, whereas those with a chronic prevention focus manifested greater right PFC activity. Eddington et al. (2007) investigated cortical activation patterns associated with promotion versus prevention goals, using a functional magnetic resonance imaging (fMRI) idiographic priming paradigm in which participants’ promotion and prevention goals were presented incidentally within a depth-of-processing task. Priming with promotion goals was associated with left PFC activation, and the observed activation was stronger for individuals with a chronic promotion focus. In a subsequent study, Eddington et al. (2009) found the same discriminant association between promotion goal priming and left PFC activation and observed that depressed individuals manifested a significantly attenuated LPFC response when primed with their own promotion goals. More recently, Shi, Ma, Wu, Wu, Wang, and Han (2016) found that larger actual:ideal self-discrepancy was associated with activations in the ventral/dorsal striatum and dorsomedial and lateral prefrontal cortices (PFCs). Similarly, Scult et al. (2017) used a monetary incentive delay task to activate reward circuitry and observed that promotion orientation was inversely correlated with ventral striatum response to gain cues.

There are a number of well-articulated models that postulate brain systems that underlie or instantiate important self-regulatory functions (e.g., Badre & Nee, 2018), including several that address personal goal pursuit. Heatherton (2011) proposed a conceptual framework for interpreting associations between different aspects of self-regulation and activity in different brain regions. This framework was elaborated by Kelley, Wagner, and Heatherton (2015) and provides a comprehensive picture of functionally connected brain regions that are engaged under different self-regulatory circumstances. For example, contemplation of personal goals has been observed to elicit activation in brain regions linked to self-reflection and/or encompassed by the default mode network (Spreng, Stevens, Chamberlain, Gilmore, & Schacter, 2010). The default mode network, which includes the medial PFC, posterior cingulate cortex, precuneus, lateral and medial temporal lobes, and posterior inferior parietal lobule, is a network of brain regions thought to be preferentially active during internally focused cognition (Buckner, Andrews-Hanna, & Schacter, 2008). Activation of regions associated with the default mode network may thus be observed in response to priming of salient promotion or prevention goals, due to the self-referential cognition required in order to define, maintain, and regulate toward such goals.

In addition to regions included in the default mode network, there is a partially overlapping network of regions along the cortical midline that has been shown to be critical for self-referential thought and therefore likely to be engaged during the process of personal goal pursuit (Northoff & Bermpohl, 2004; Qin & Northoff, 2011). This *cortical midline structures model* includes the orbital and dorsomedial PFC, the anterior cingulate cortex, and the posterior cingulate cortex including the precuneus (Northoff & Bermpohl, 2004). These structures are associated with the representation and processing of self-relevant stimuli (Beer & Ochsner, 2006; Northoff & Bermpohl, 2004), suggesting their potential relevance for identifying broad patterns of activation following promotion/prevention priming.

Most recently, Langner, Leiberg, Hoffstaedter, and Eickhoff (2018) conducted two meta-analyses of human neuroimaging studies on emotion or action control. Their conceptual framework emerged in part from a study by Murray, Debbane, Fox, Bzdok, and Eickhoff (2015) that combined task-dependent meta-analytic connectivity modeling with task-independent resting-state connectivity analysis and focused on similarities and distinctions between self-processing and other processing. Murray and colleagues found two functionally connected subnetworks, one preferentially activated during self-relevant processing and the other during other-relevant processing. Langer and colleagues summarized a partially distinct set of studies and reported two fronto-parieto-insular networks with limited overlap (bilateral anterior insula, posteromedial PFC, and right temporo-parietal junction). The investigators went on to postulate a core brain/behavior system for implementing self-control across both emotion and goal-directed action but also noted that there were distinctions on functional connectivity analyses between components of the system related to affective versus executive aspects of self-control.

To date, several neuroimaging studies of promotion and prevention focus have found activation in one or more of the aforementioned self-regulation-relevant regions. Johnson et al. (2006) used fMRI to investigate differences in neural activation in response to promotion and prevention goals in contrast with nonself-relevant items. Their results indicated that reflecting on promotion/prevention goals was associated with greater activity in both the medial/anterior cingulate cortex and the posterior cingulate cortex/precuneus. Moreover, they found that the frontal regions were relatively more active in response to reflecting on promotion goals, whereas the posterior regions were more active in reflecting on prevention goals. Packer and Cunningham (2009) investigated whether temporal proximity to a promotion or prevention goal altered neural activation. In addition to observing differences based on how near or far a hypothetical goal was in time, they again observed activation in the medial PFC, anterior cingulate cortex, and the precuneus; however, they did not observe the same dissociation between promotion and prevention goals as was reported by Johnson et al. (2006). As noted above, Shi et al. (2016) found that actual:ideal self-discrepancy predicted activation in the dorsal medial and lateral PFCs.

Strauman et al. (2013) used a different task-based fMRI approach to further elucidate the neural correlates of promotion and prevention goal pursuit. Applying a rapid masked priming technique in which participants were exposed subliminally to their own previously assessed promotion and prevention goals, the authors observed distinct patterns of neural activation associated with promotion versus prevention goals. Promotion priming led to activation in frontal and occipital regions as well as caudate and thalamus, whereas prevention priming was associated with activation in precuneus and posterior cingulate cortex. Individual differences in chronic dysphoric/anxious affect and in regulatory focus predicted differential activation following promotion versus prevention priming. The regions activated in response to promotion and prevention goals mapped broadly onto the cortical midline structures network. However, Strauman et al. (2013) did not systematically vary the extent to which the goals being used as priming stimuli were perceived by participants as cues for success (i.e., goals they believed they were attaining or had attained) versus failure (i.e., goals they believed they had not attained). According to RFT, this dimension of goal representation and goal pursuit processing should have profound motivational and affective consequences, which in turn could be associated with distinct neural activation patterns.

To our knowledge, no study has systematically manipulated both dimensions that SDT and RFT hypothesize as critical for determining the motivational and affective significance of goal activation: *domain of goal* (i.e., promotion/ideal vs. prevention/ought) and *attainment status* (i.e., one’s perceived behavior/attributes matching versus mismatching a motivationally significant goal). In the present study, we asked two related questions: first, what patterns of neural activity are associated with priming of promotion versus prevention goals also considering the perceived attainment status of each goal; and second, to what extent are the observed patterns of neural activity for each type of goal related to a self-report index of individual differences in chronic regulatory focus.

We chose to examine the data using two complementary analytic approaches: a standard general linear model (GLM) whole-brain analysis looking for statistically significant regions of activation associated with a particular theory-based contrast (e.g., promotion match goal priming vs. control priming) and a task-based functional connectivity analysis looking for common versus unique patterns of activation across the domain of goal and attainment status priming manipulations. The standard GLM analysis allowed us to compare our findings directly with published studies in a larger sample, whereas the functional connectivity analysis was intended to examine the overlap between neural activations following promotion versus prevention goal priming and existing cognitive neuroscience models of self-regulation. Following the recommendations of Beckmann and Smith (2005) and Guo and Pagnoni (2008), tensorial probabilistic independent component analysis (TICA) was chosen for the task-based functional connectivity analysis because it affords two primary benefits when compared with seed-based psychophysiological interaction analysis. First, it identifies a set of statistically independent components, a subset of which can then be selected for further examination based on a priori criteria. This process reduces the number of comparisons and increases statistical power to detect differences across priming conditions. Second, because TICA extracts components without a model or spatial parameters and then tests for statistically significant associations with task-based model parameters, it provides an optimal blend of exploratory and hypothesis-testing approaches to functional connectivity – particularly valuable in this context because of the likelihood that activation across the priming conditions would have both common and unique components. In the following sections, we describe the methods and findings following the reporting recommendations of Nichols et al. (2016, 2017).

## 1. Method

### 1.1 Participants

A total of 55 participants were approached through two sources (see below), and all 55 responded in the affirmative and then gave informed consent and completed the study. A subset of participants (*N* = 7) was excluded from analyses due to either excessive head motion during scanning (≥3.0 mm; *N* = 2), exceeding threshold on one or more quality control measures (see below; *N* = 3), or technical issues while scanning (*N* = 2), leaving a final sample of 48 participants with complete data (28 females and 20 males). Participants had a mean age of 28.32 ± 11 years (range = 18–60), and all were right-handed as per self-report. Of these 48 participants, 32 were Caucasian, 6 were African-American, and 10 were Asian. Participants were recruited either through the participant pool at the Duke Interdisciplinary Institute for Social Psychology Lab or through the Duke Neurogenetics Study, a longitudinal investigation in which participants had agreed to be recontacted for further research participation. Informed consent was obtained prior to participation. Participants were compensated $20 per hour.

### 1.2 Procedure

The study consisted of two sessions conducted approximately 4 weeks apart. In the first session, participants completed the Regulatory Focus Questionnaire (RFQ; Higgins et al., 2001) and the Computerized Goal Assessment task (Shah & Higgins, 2001), which provided the data needed to create the priming stimuli and the measures of individual differences in promotion and prevention orientation. In the second session, participants were scanned while completing an idiographic subliminal priming task, debriefed, and compensated for their time. All procedures were approved by the Duke University Institutional Review Board.

The RFQ is a self-report measure designed to assess the degree to which individuals are oriented toward promotion and/or prevention. The RFQ used in this study contained 12 Likert style items divided into two subscales measuring the extent to which an individual believes they have been successful in attaining promotion or prevention goals. The subscales include items such as: “I feel like I have made progress toward being successful in my life” (promotion success) and “Not being careful enough has gotten me in trouble at times” (prevention success, reverse-scored). Each subscale has an internal consistency (coefficient alpha) of .75 or higher and a 2-month test–retest reliability (Pearson correlation) of .79 or higher.

The Computerized Goal Assessment task (Shah & Higgins, 2001) is a computer-administered measure of self-reported attributes that describe one’s ideal and ought selves. Participants were first given definitions of what ideal and ought attributes are (i.e., “attributes of the kind of person you wish or desire to be” and “attributes of the person you believe it is you duty or responsibility to be,” respectively). They were then serially prompted to think of six attributes to describe their ideal self and six attributes to describe their ought self, in alternating order. Participants were discouraged from listing more than one word for each response, from repeating responses, or from listing synonyms. After each response, participants were then asked to rate the attribute using a scale from one (not at all) to seven (extremely) for the following question: “How far are you from possessing this attribute?”. Four attributes were selected from each category (i.e., ideal and ought) for each participant to be used as self-relevant priming stimuli in the subsequent scanning session: the two most highly “matched” attributes (i.e., the attributes they rated as being closest to possessing on the 7-point scale) and the two most highly “mismatched” attributes (i.e., the attributes they rated as being farthest from possessing on the 7-point scale). Four yoked-control attributes were also selected from the responses of another participant selected at random (two ideal adjectives and two ought adjectives); these words were semantically unrelated to the attributes listed by the participant.

**Figure 1.**
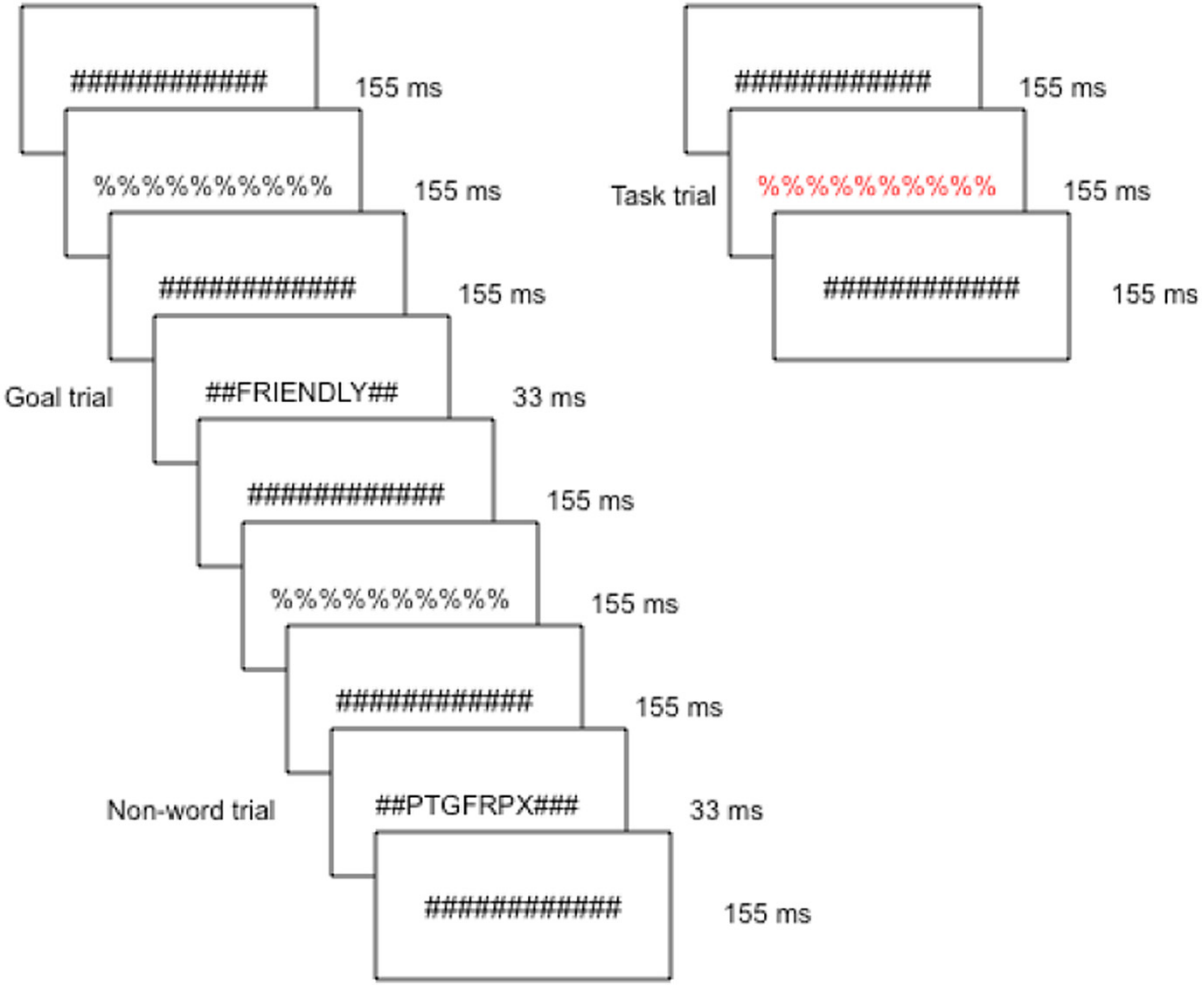
Schematic of the experimental task. The sequence for an individual trial consisted of alternating pound signs and percent signs, in between which a word or nonword was inserted. Incidental to the promotion goal, prevention goal, and yoked-control priming stimuli that were inserted throughout the run, visible colored stimuli were displayed to which participants were instructed to press a button as quickly as possible.

In a subsequent session, participants then completed an event-related rapid masked priming fMRI task (Strauman et al., 2013; see Figure 1). In this task, modeled on the task developed by Diaz and McCarthy (2007), participants were exposed to a continuous series of rapidly presented, masked visual stimuli. These stimuli included the participants’ previously listed ideal and ought attributes, the ideal and ought attributes of another participant as a yoked-control comparison condition, and nonword letter strings. All goal words and all yoked-control words were positively valenced trait attributes; nonwords were random consonant strings, each 4–10 characters in length. Each letter string was padded with pound signs so that letters were centered and each stimulus was 12 characters in length to ensure that the same amount of the visual field was occupied at any given time during the experiment.

A critical stimulus occurred approximately every 500 ms within the constantly changing visual display; the duration of critical stimulus presentation was 33 ms. Trials primarily consisted of masked nonwords, and masked goal words or yoked-control words were presented once every 12–15 s. All word and nonword strings were preceded and followed by longer duration pound sign strings, which served as pattern masks. The pound sign strings in turn alternated with percent sign strings such that participants experienced a continuously changing visual stream. As an attentional control, participants were instructed to make a button-press response when they detected a pound sign string presented in red font. These target events occurred infrequently (mean interval = 25 s) and for a longer duration (155 ms) and were not in close temporal proximity to the masked attribute trials. Participants were not told that either words or nonword strings would be presented.

The task was divided into four runs of 6 min each. The run conditions were as follows: ideal match (ideal attributes rated as closest to possessing), ideal mismatch (ideal attributes rated as farthest from possessing), ought match (ought attributes rated as closest to possessing), and ought mismatch (ought attributes rated as farthest from possessing). Each run also included yoked-control attributes (goal attributes selected from a different subject that were not semantically related to any of the target participant’s goals) to allow for creation of contrasts. Run order was randomized across participants to control for any order effects, and initial statistical analyses detected no statistically significant effect of run order. Stimuli were presented using CIGAL, an in-house data presentation software program (Voyvodic, 1999). All stimuli were displayed on MRI-compatible LCD goggles.

#### 1.2.1 Image acquisition

Neuroimaging data were acquired on a 3.0 Tesla GE Signa EXCITE HD system (Waukesha, WI, USA) at Duke’s Brain Imaging and Analysis Center (www.biac.duke.edu). Four initial RF excitations were acquired and discarded to achieve steady-state equilibrium. For each subject, 720 functional images (180 per run) with 34 interleaved 4-mm-thick slices were acquired in the oblique axial plane using a T2* -weighted sense spiral sequence (TR/TE = 2000/30 ms, voxel dimensions = 3.8 × 3.8 × 3.8 mm, flip angle = 60**°**, field of view = 240 mm, matrix size = 64 × 64). Prior to functional image collection, a T1-weighted coplanar structural scan with 34.4-mm slices (TR/TE = 7600/30 ms, flip angle = 12**°**; field of view = 240 mm, matrix size = 256 × 256) was acquired parallel to the AC–PC line for spatial normalization and coregistration of the functional data to a standard atlas.

#### 1.2.2 Quality control

Prior to preprocessing the fMRI data, we obtained three measures of data quality and excluded subjects with extreme values. First, we estimated the average signal-to-fluctuation-noise ratio for each subject, defined as the mean of the signal across time divided by the standard deviation of the signal across time. Second, we computed the mean volume-to-volume head motion (i.e., displacements relative to the preceding time point in units of millimeter) for each subject. Third, using an FMRIB’s Software Library (FSL) tool called *fsl_motion_outliers*, we identified outlier volumes in the functional data by examining the root-mean-square error (RMSE) of each volume relative to the reference volume (with the middle time point used as reference). A volume was judged to be an outlier if its RMSE amplitude exceeded the 75th percentile plus the value of 150% of the interquartile range of RMSE for all volumes in a run (representing a standard boxplot threshold). We excluded subjects where any of the three quality control measures was extreme (beyond the upper or lower 5th percentile). This procedure resulted in excluding the data from three participants.

#### 1.2.3 fMRI data preprocessing

The functional data were preprocessed using FMRI Expert Analysis Tool within FSL (Oxford University; www.fmrib.ox.ac.uk/fsl). The following steps were utilized: motion correction by coregistration of all volumes to a reference volume using MCFLIRT nonbrain structures removal using Brain Extraction Tool spatial smoothing using a Gaussian kernel of 8.0-mm Full Width Half Maximum; grand mean intensity normalization of the entire 4D dataset by a single multiplicative factor; and high-pass temporal filtering (Gaussian-weighted least squares straight line fitting, 50-s cutoff).

#### 1.2.4 GLM analyses

Whole-brain GLM-based analyses were conducted to identify regions activated by promotion and prevention goal priming for both match and mismatch conditions. The GLM analysis methods closely followed past procedures (Strauman et al., 2013) in order to determine whether previous findings were replicated in the present sample. All statistical images were thresholded using clusters determined by *Z* > 2.3 and a whole-brain corrected cluster significance threshold of *p* < .05, supplemented by the nonparametric permutation method recommended by Eklund, Nichols, & Knutson (2016). First, denoized functional data were analyzed using a GLM with local autocorrelation correction (Woolrich, Ripley, Brady, & Smith, 2001). For each run, separate regressors were created for the goal priming words, the yoked-control words, and the nonword stimuli. A nuisance regressor modeled the button-press response component of the task. All regressors consisted of unit impulses convolved with a canonical hemodynamic response function. Four contrasts were created for each participant: (1) ideal match versus yoked-control, (2) ideal mismatch versus yoked-control, (3) ought match versus yoked-control, and (4) ought mismatch versus yoked-control. Data were combined across subjects using a mixed-effects model (Beckmann, Jenkinson, & Smith, 2003; Woolrich, Behrens, Beckmann, Jenkinson, & Smith, 2004). Mixed-effects models were then used to obtain statistical tests for whether the two individual difference measures (promotion and prevention success) were associated with activation following goal priming (again using the four contrasts created for each participant). Analyses of the priming condition contrasts and the individual differences measures were carried out using FLAME (FMRIB’s Local Analysis of Mixed Effects) – stage 1 (Beckmann et al., 2003; Woolrich et al., 2004), consisting on a mixed-effects (fixed and random effects) approach using Bayesian estimation techniques. To account for the likelihood that some brain regions might be responsive to particular kind of priming stimuli only among individuals characterized by high or low levels of chronic perceived success in personal goal pursuit, the individual difference measures were included in the whole-brain analyses. Throughout the analyses, no statistically significant main effects or interactions involving participant gender or run order were identified. Therefore, none of the analyses reported below include gender or run order as a between-subject effect.

#### 1.2.5 Independent component analyses

TICA is a technique that extends single-session independent component analysis (ICA) to higher dimensions as an approach to multisession/multisubject task-based fMRI data analysis. Our TICA analysis was performed with Multivariate Exploratory Linear Decomposition into Independent Components (MELODIC) Version 3.14 from the FSL (www.fmrib.ox.ac.uk/fsl). Four TICA analyses were conducted, one for each priming condition contrast corresponding to each of the GLM analyses for priming condition. Within each analysis, all subjects were time concatenated for a single 48-run (48 subjects) group ICA, using the same preprocessed functional data that were entered into the GLM analyses. All subjects’ data were spatially normalized to an anatomical MNI standard template using a 12-parameter affine registration implemented in FLIRT). The voxel BOLD times series were demeaned, variance normalized, and whitened. The number of independent components extracted by MELODIC was estimated using a Bayesian approach. Using Probabilistic Independent Component Analysis (Beckmann & Smith, 2005), the whitened time data were projected onto a multidimensional subspace. Fixed-point iteration optimization was used to decompose the projected data into independent vector sets that account for variability in temporal, spatial, and subject domains. The spatial components were normalized by the variance of the residuals, and a mixture model was fit to the obtained intensity histograms to determine a statistical threshold (Beckmann & Smith, 2005).

Within each TICA analysis, we included a regressor for the contrast of interest (e.g., ideal/match vs. yoked-control) as well as regressors for the RFQ promotion and prevention scores. For each analysis, we used the following a priori criteria to determine the number of components for further examination (following the recommendations of Najeed & Avison, 2014): (1) the component needed to account for at least 5.0% of the total variance within that analysis, (2) the component did not resemble artifact or noise upon visual inspection, particularly with reference to the timing of activation peaks in comparison with the contrast regressors, (3) the overall *F* value for the set of three theory-based regressors (contrast of interest, RFQ promotion score, and RFQ prevention score) needed to meet statistical significance at *p* < .01, and (4) at least one of the three regressors needed to meet statistical significance at *p* < .01. These more lenient statistical criteria were used in order to maximize the exploratory power of the TICA analyses, given the more conservative nature of this analytic method compared with GLM-based analyses (Beckmann & Smith, 2005).

#### 1.2.6 Statistical power

Power analysis was performed following the recommendations of Geuter et al. (2018) and Mumford (2012). Power estimation was based on the findings of Strauman et al. (2013) who used the identical idiographic goal priming procedure and conducted whole-brain GLM analyses with priming condition/contrast, a within-subject manipulation, as the primary predictor of interest. Strauman et al. observed a medium-to-large effect size (Cohen’s *d* = .68) based on a null-hypothesis test of the ideal versus control priming contrast of parameter estimates. Based on those data, the present study (with a final *N* of 48 participants) had 71% power to detect a similar sized effect in whole-brain GLM analyses.

## 2. Results

### 2.1 GLM analyses: Activation in response to each goal priming condition

In order to identify activation in response to being primed by matching or mismatching promotion or prevention goals, BOLD signal change in response to each type of priming word was determined contrasted with activation in response to yoked-control words within the same run. To account for the likelihood that some regions might be responsive to particular kinds of priming stimuli only among individuals characterized by high or low levels of chronic perceived success in personal goal pursuit, the promotion and prevention success scores were included in these analyses as covariates.

#### 2.1.1 Ideal match versus yoked-control contrast

Table 1 lists the regions showing statistically significant activation for the ideal match versus control contrast. For the contrast main effect, there was a single cluster comprised of regions in bilateral parietal lobe (primarily bilateral precuneus, Brodmann area (BA) 7). In addition, scores on the promotion success subscale were significantly positively correlated with activation on the ideal match versus control contrast in two distinct clusters. The first cluster was distributed across the frontal, temporal, and occipital lobes including left middle frontal gyrus (BA 9), left inferior and superior temporal gyrus (BA 20 and 38), and left fusiform gyrus (BA 10). The second cluster was distributed across the temporal, occipital, and parietal lobes and included right inferior and middle temporal gyrus and precuneus (BA 7, 20, and 21) and right lateral occipital cortex (BA 19).

**Table 1.** Regions associated with the ideal/match versus yoked-control contrast in GLM analysis

Probabilistic Anatomical Label	Brodmann Area	*x*	*y*	*z*	*Z*-stat	Cluster Volume (mm^3^) / (*P*)
Main effect for ideal/match versus control priming						
Right parietal lobe, precuneous cortex (65%)	7	10	–66	62	4.41	4470
Left parietal lobe, precuneous cortex (55%)	7	–6	–66	66	4.02	(*P* < .01)
Left parietal lobe, precuneous cortex (39%)	7	–4	–62	64	3.80	
Right parietal lobe, precuneous cortex (45%)	7	2	–62	54	3.42	
Right parietal lobe, precuneous cortex (38%)	7	6	–78	50	3.40	
Left parietal lobe, precuneous cortex (35%)	7	–2	–60	64	3.38	
Positive correlation with RFQ promotion success scale						
Left frontal lobe, middle frontal gyrus (41%)	9	–38	30	32	4.18	12021
Left temporal lobe, temporal pole (43%)	38	–46	10	–24	4.01	(*P* < .001)
Left temporal lobe, inferior temporal gyrus (62%)	20	–46	–36	–18	3.89	
Left occipital lobe, fusiform gyrus (91%)	18	–24	–90	–18	3.73	
Left frontal lobe, middle frontal gyrus (73%)	9	–30	36	30	3.65	
Left frontal lobe, middle frontal gyrus (55%)	9	–28	40	32	3.64	
Right temporal lobe, middle temporal gyrus (69%)	21	58	–28	–14	4.08	10138
Right temporal lobe, inferior temporal gyrus (85%)	37	54	–50	–16	3.81	(*P* < .001)
Right occipital lobe, lateral occipital cortex (95%)	19	46	–78	2	3.73	
Right occipital lobe, lateral occipital cortex (40%)	19	48	–78	14	3.66	
Right occipital lobe, lateral occipital cortex (78%)	7	40	–64	52	3.64	
Right temporal lobe, inferior temporal gyrus (43%)	20	54	–32	–16	3.41	

Regions manifesting statistically significant activation in the ideal/match versus yoked-control contrast. *N* = 48. Coordinates of the top six local maxima within each cluster of activation are in MNI space. Probabilistic labels reflect the likelihood that a coordinate belongs to a given region; only the label with the highest probability is shown.

#### 2.1.2 Ideal mismatch versus yoked-control contrast

Table 2 lists the regions showing statistically significant activation for the ideal mismatch versus control contrast. For contrast main effect, there was a single cluster comprised of regions in bilateral occipital lobe (primarily bilateral cuneus, BA 18). In addition, scores on the promotion success subscale were significantly associated (and positively correlated) with activation on the ideal mismatch versus control contrast in a single cluster which was centered in the left frontal lobe (specifically left middle frontal gyrus and inferior frontal gyrus, BA 9 and 45/46/47, respectively).

**Table 2.** Regions associated with the ideal/mismatch versus yoked-control contrast in GLM analysis

Probabilistic Anatomical Label	Brodmann Area	*x*	*y*	*z*	*Z-stat*	Cluster Volume (mm^3^) / (*P*)
Main effect for ideal/mismatch versus control priming						
Right occipital lobe, occipital pole (70%)	18	14	–98	10	4.05	5879
Left occipital lobe, fusiform gyrus (81%)	18	–22	–76	–2	4.03	(*P* < .01)
Left occipital lobe, occipital pole (90%)	18	–10	–96	12	3.88	
Left occipital lobe, occipital pole (93%)	18	–8	–98	10	3.82	
Right occipital lobe, intracalcarine cortex (34%)	18	16	–84	12	3.81	
Right occipital lobe, intracalcarine cortex (79%)	18	6	–76	6	3.74	
Positive correlation with RFQ promotion success						
Left frontal lobe, frontal pole (78%)	47	–46	36	–16	4.67	5458
Left frontal lobe, inferior frontal gyrus (66%)	9	–40	16	24	4.29	(*P* < .01)
Left frontal lobe, inferior frontal gyrus (48%)	45	–50	34	2	3.89	
Left frontal lobe, middle frontal gyrus (77%)	6	–42	6	48	3.65	
Left frontal lobe, inferior frontal gyrus (34%)	46	–50	26	12	3.50	
Left frontal lobe, inferior frontal gyrus (45%)	46	–42	18	22	3.48	

Regions manifesting statistically significant activation in the ideal/mismatch versus yoked-control contrast. *N* = 48. Coordinates of the top six local maxima within each cluster of activation are in MNI space. Probabilistic labels reflect the likelihood that a coordinate belongs to a given region; only the label with the highest probability is shown.

#### 2.1.3 Ought match versus yoked-control contrast

Table 3 lists the regions showing statistically significant activation for the ought match versus control contrast. For the contrast main effect, two significant clusters were found. The first cluster was distributed across the frontal lobes and included right middle and superior frontal gyrus (BA 8/9/10) and left middle frontal gyrus (BA 6). The second cluster was found in the parietal lobes and included right and left supramarginal gyrus and right angular gyrus (BA 7 and 40). There were no regions of activation for the ought match versus control contrast which were significantly correlated with scores on either the promotion or prevention success subscales.

**Table 3.** Regions associated with the ought/match versus yoked-control contrast in GLM analysis

Probabilistic Anatomical Label	Brodmann Area	*x*	*y*	*z*	*Z*-stat	Cluster Volume (mm^3^)/(*P*)
Main effect for ought/match versus control priming						
Right frontal lobe, frontal pole (95%)	46	44	44	16	5.53	9460
Right frontal lobe, middle frontal gyrus (77%)	8	42	24	42	4.83	(*P* < .001)
Left frontal lobe, middle frontal gyrus (64%)	6	–48	8	44	4.39	
Right frontal lobe, frontal pole (75%)	10	32	60	10	4.13	
Left frontal lobe, superior frontal gyrus (35%)	6	–24	–2	48	3.85	
Right frontal lobe, middle frontal gyrus (91%)	9	52	12	36	3.81	
Right parietal lobe, angular gyrus (46%)	40	52	–50	46	4.04	6288
Right parietal lobe, angular gyrus (63%)	40	56	–52	38	4.04	(*P* < .01)
Right parietal lobe, angular gyrus (58%)	40	46	–48	54	3.96	
Right parietal lobe, angular gyrus (66%)	40	60	–46	32	3.91	
Left parietal lobe, lateral occipital cortex (61%)	7	–36	–68	46	3.86	
Left parietal lobe, supramarginal gyrus (47%)	40	–46	–48	46	3.75	

Regions manifesting statistically significant activation in the ought/match versus yoked-control contrast. *N* = 48. Coordinates of the top six local maxima within each cluster of activation are in MNI space. Probabilistic labels reflect the likelihood that a coordinate belongs to a given region; only the label with the highest probability is shown.

#### 2.1.4 Ought mismatch versus yoked-control contrast

Table 4 lists the regions showing statistically significant activation for the ought mismatch versus control contrast. For the contrast main effect, one significant cluster was found. The cluster included maxima in bilateral caudate as well as bilateral occipital lobe, specifically left and right intracalcarine cortex (BA 18). There were no regions of activation for the ought match versus control contrast which were significantly correlated with scores on either the promotion or prevention success subscales.

**Table 4.** Regions associated with the ought/mismatch versus yoked-control contrast in GLM analysis

Probabilistic Anatomical Label	Brodmann Area	*x*	*y*	*z*	*Z*-stat	Cluster Volume (mm^3^)/(*P*)
Main effect for ought/mismatch versus control priming						
Left caudate (82%)		–12	12	16	4.96	13566
Left occipital lobe, intracalcarine cortex (86%)	18	–12	–74	4	4.83	(*P* < .001)
Right occipital lobe, intracalcarine cortex (82%)	18	10	–74	6	4.70	
Left occipital lobe, fusiform gyrus (97%)	18	–18	–76	–10	4.22	
Right caudate (99%)		12	12	12	4.04	
Left caudate (99%)		–14	16	8	3.95	

Regions manifesting statistically significant activation in the ought/mismatch versus yoked-control contrast. *N* = 48. Coordinates of the top six local maxima within a single cluster of activation are in MNI space. Probabilistic labels reflect the likelihood that a coordinate belongs to a given region; only the label with the highest probability is shown.

### 2.2 TICA analyses: Independent components associated with priming condition and/or RFQ scores

For each of the four separate TICA analyses (each corresponding to one of the four GLM analyses by priming condition), we applied the component selection criteria described above to identify components that accounted for sufficient variance and were associated with either the contrast of interest *per se* or to the RFQ promotion or prevention scores.

#### 2.2.1 Components associated with the ideal match versus yoked-control contrast

**Figure 2.**
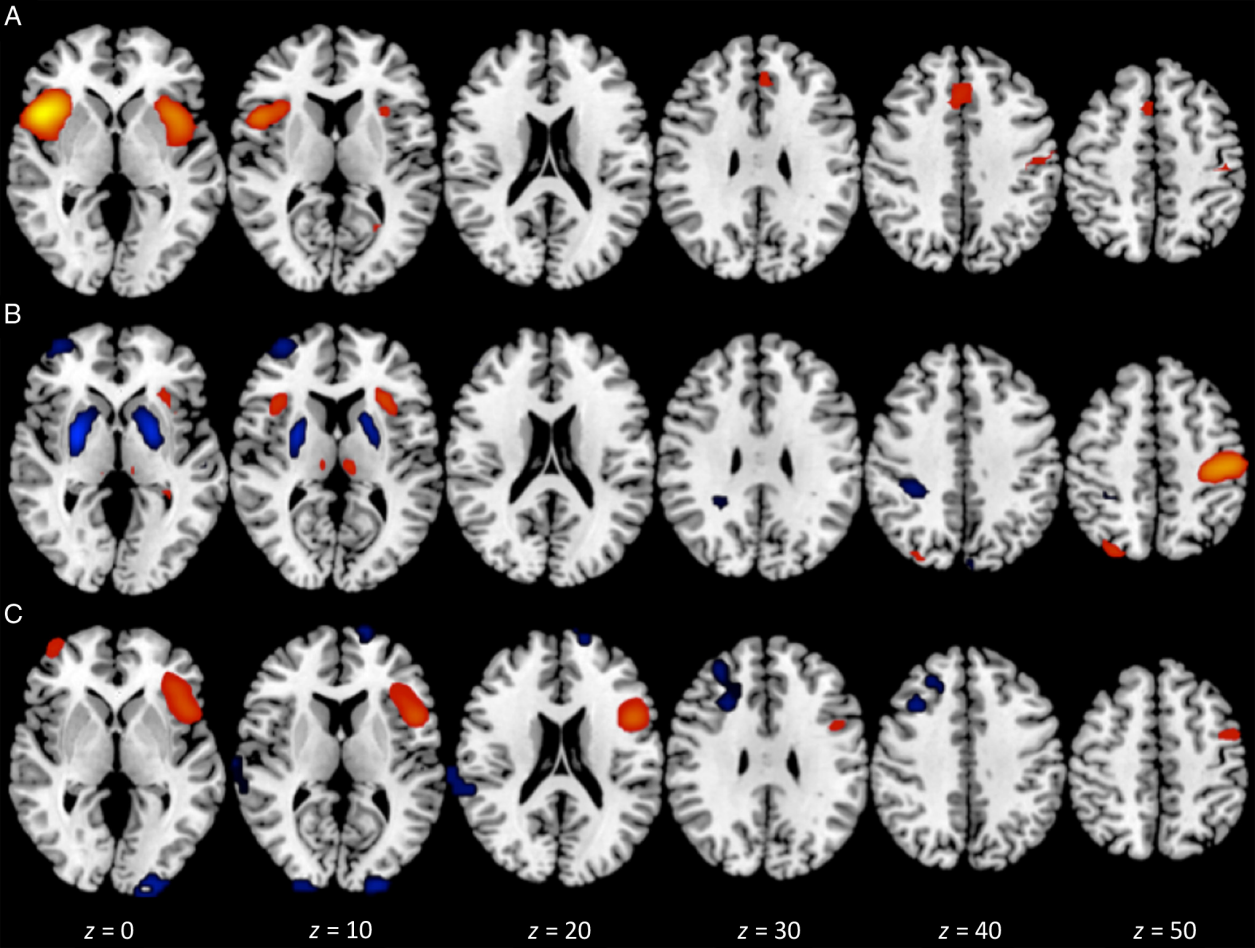
Spatial maps for components of interest in the ideal match versus control contrast analysis from TICA. The three components shown were selected via a priori criteria and are ordered by decreasing percent of total variance accounted for. Each map depicts axial views at *z* = 0, 10, 20, 30, 40, and 50 mm and is thresholded at *Z* ±2.30. Red/yellow signifies areas of significant activation; blue signifies areas of significant deactivation. Component A: 6.91% of variance, associated with both the contrast and promotion success scores. Component B: 5.92% of variance, associated with the contrast only. Component C: 5.11% of variance, associated with promotion success scores only.

We found three components of interest related to this priming condition. The first component accounted for 6.91% of total variance, had an excellent overall model fit, *F*(3, 45) = 26.00, *p* < .0001, and was significantly associated with both the contrast *per se* and with scores on the promotion success scale, *Z* = 6.58 (*p* < .001) and 2.22 (*p* < .02), respectively. The loci of greatest activation were in bilateral insula (BA 13) and bilateral precentral gyrus (BA 44), with no regions showing significant deactivation. The second component accounted for 5.92% of total variance, had an excellent overall model fit, *F*(3, 45) = 17.21, *p* < .0001, and was significantly associated with the contrast, *Z* = 5.67, *p* < .001. The loci of greatest activation were in left parietal lobe/postcentral gyrus (BA 3) and bilateral insula (BA 13). In addition, significant control > ideal-match activation was found in right superior frontal gyrus (BA 10) and bilateral putamen. The third component accounted for 5.11% of total variance, had a good overall model fit, *F*(3, 45) = 5.65, *p* < .01, and was significantly associated with scores on the promotion success scale, *Z* = 2.77, *p* < .01. The loci of greatest activation were in left middle frontal gyrus (BA 9 and 6), left superior frontal gyrus (BA 6), and right occipital gyrus (BA 19). In addition, significant deactivation was found in right middle frontal gyrus (BA 9). Figure 2 presents visual summaries of each of the three components for the ideal match TICA analysis.

#### 2.2.2 Components associated with the ideal mismatch versus yoked-control contrast

**Figure 3.**
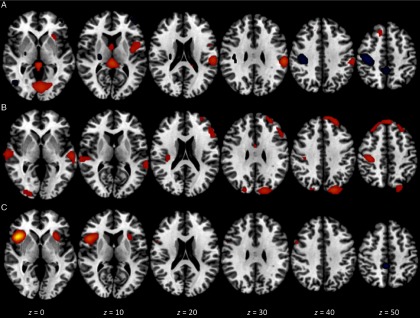
Spatial maps for components of interest in the ideal mismatch versus control contrast analysis from TICA. The three components shown were selected via a priori criteria and are ordered by decreasing percent of total variance accounted for. Each map depicts axial views at *z* = 0, 10, 20, 30, 40, and 50 mm and is thresholded at *Z* ±2.30. Red/yellow signifies areas of significant activation; blue signifies areas of significant deactivation. Component A: 9.48% of variance, associated with both the contrast and promotion success scores. Component B: 8.30% of variance, associated with both the contrast and promotion success scores. Component C: 6.29% of variance, associated with both the contrast and promotion success scores.

We found three components of interest related to this contrast. The first component accounted for 9.48% of total variance, had an excellent overall model fit, *F*(3, 45) = 62.33, *p* < .0001, and was significantly associated with both the contrast *per se* and with scores on the promotion success scale, *Z* = 8.50 (*p* < .001) and 2.06 (*p* < .02), respectively. The loci of greatest activation were in bilateral occipital lobe (BA 18), left precentral gyrus (BA 6), and left parietal lobe (BA 40). In addition, significant deactivation was found in right parietal lobe (BA 3). The second component accounted for 8.30% of total variance, had an excellent overall model fit, *F*(3, 45) = 44.18, *p* < .0001, and was significantly associated with both the contrast *per se* and with scores on the promotion success scale, *Z* = 7.48 (*p* < .001) and 3.77 (*p* < .001), respectively. The loci of greatest activation were in bilateral temporal lobe (BA 22), bilateral occipital lobe (BA 18), bilateral superior frontal gyrus (BA 8), left middle frontal gyrus (BA 9), and right precentral gyrus (BA 4). No significant deactivation was observed. The third component accounted for 6.29% of total variance, had an excellent overall model fit, *F*(3, 45) = 14.74, *p* < .0001, and was significantly associated with both the contrast *per se* and with scores on the promotion success scale, *Z* = 5.41 (*p* < .001) and 2.08 (*p* < .02), respectively. The loci of greatest activation were in bilateral insula (BA 13). In addition, significant deactivation was found in bilateral precuneus (BA 7). Figure 3 presents visual summaries of each of the three components for the ideal mismatch TICA analysis.

#### 2.2.3 Components associated with the ought match versus yoked-control contrast

**Figure 4.**
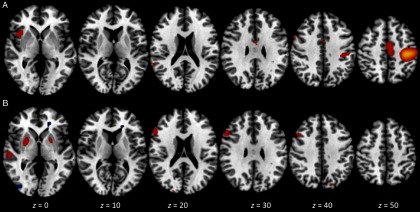
Spatial maps for components of interest in the ought match versus control contrast analysis from TICA. The two components shown were selected via a priori criteria and are ordered by decreasing percent of total variance accounted for. Each map depicts axial views at *z* = 0, 10, 20, 30, 40, and 50 mm and is thresholded at *Z* ±2.30. Red/yellow signifies areas of significant activation; blue signifies areas of significant deactivation. Component A: 12.40% of variance, associated with the contrast only. Component B: 6.01% of variance, associated with the contrast only.

We found two components of interest related to this contrast. The first component accounted for 12.40% of total variance, had an excellent overall model fit, *F*(3, 45) = 17.83, *p* < .0001, and was significantly associated with the contrast, *Z* = 5.76, *p* < .001. The loci of greatest activation were in left precentral gyrus (BA 3 and 4) and bilateral medial frontal gyrus (BA 6), with no regions showing significant deactivation. The second component accounted for 6.01% of total variance, had a good overall model fit, *F*(3, 45) = 4.98, *p* < .01, and was significantly associated with the contrast, *Z* = 3.82, *p* < .001. The loci of greatest activation were in right superior frontal gyrus (BA 8), bilateral insula (BA 13), and right frontal gyrus (BA 6). In addition, significant deactivation was found in right occipital gyrus (BA 18). Figure 4 presents visual summaries of each of the two components for the ought match TICA analysis.

#### 2.2.4 Components associated with the ought mismatch versus yoked-control contrast

**Figure 5.**
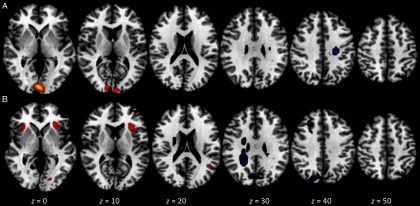
Spatial maps for components of interest in the ought mismatch versus control contrast analysis from TICA. The two components shown were selected via a priori criteria and are ordered by decreasing percent of total variance accounted for. Each map depicts axial views at *z* = 0, 10, 20, 30, 40, and 50 mm and is thresholded at *Z* ±2.30. Red/yellow signifies areas of significant activation; blue signifies areas of significant deactivation. Component A: 9.87% of variance, associated with the contrast only. Component B: 6.75% of variance, associated with the contrast only.

Using our selection criteria, we found two components of interest related to this contrast. The first component accounted for 9.87% of total variance, had an excellent overall model fit, *F*(3, 45) = 32.18, *p* < .0001, and was significantly associated with the contrast, *Z* = 7.11, *p* < .001. The loci of greatest activation were in bilateral occipital lobe (BA 18). There was also significant deactivation in left frontal gyrus (BA 6). The second component accounted for 6.75% of total variance, had a good overall model fit, *F*(3, 45) = 4.46, *p* < .01, and was significantly associated with the contrast, *Z* = 3.34, *p* < .001. The loci of greatest activation were in bilateral insula (BA 13) and left temporal gyrus (BA 39). In addition, significant deactivation was found in left middle frontal gyrus (BA 10), bilateral occipital lobe (BA 18), and right superior temporal gyrus (BA 42). Figure 5 presents visual summaries of each of the two components for the ought mismatch TICA analysis.

## 3. Discussion

To our knowledge, the present study was the first to use an experimental design to investigate the neural correlates of promotion and prevention goal priming, while also manipulating the theoretically important distinction between perceived goal attainment versus nonattainment. We predicted significant activation for idiographic goal priming above and beyond activation to yoked-control words and that these activation patterns would differ based on goal type (promotion/ideal vs. prevention/ought) and perceived goal attainment (“match” vs. “mismatch”). We also hypothesized that variability in those activation patterns would be associated with self-reported individual differences in history of success pursuing such goals. We used two complementary analytic approaches: a GLM-based whole-brain analysis looking for statistically significant regions of activation associated with each theory-based contrast and a TICA-based functional connectivity across the four goal priming manipulations.

Our hypotheses were largely confirmed. First, priming of both promotion and prevention goal attributes was associated with significant activation over and above the activation seen in response to yoked-control words, which were personal goals from a different participant without motivational significance for the target participant. Second, each priming condition was associated with both common and distinct areas of activation. Third, for promotion goals (both ideal/match and ideal/mismatch), the patterns of activation for goal priming were also modulated by individual differences in self-reported promotion goal pursuit success (but not by prevention success).

Based on a larger sample size, a much greater number of priming trials per participant, and the use of two complementary data analysis techniques, we were able to identify a greater number of brain regions associated with each priming condition compared with previous studies using idiographic promotion and prevention goal priming (Eddington et al., 2007, 2009; Strauman et al., 2013). The rapid masked priming procedure we adapted from Diaz and McCarthy (2007) had several advantages. One was that because the priming itself was both rapid and masked, it was unlikely that the stimuli were consciously perceived; this helped to reduce any desirability effects or demand characteristics in relation to the specific goals being primed (whether personally relevant or yoked-control). Another was that we could increase substantially the number of priming trials without increasing the overall time required to complete the task. Perhaps most importantly, the findings of Diaz and McCarthy (2007) – specifically, that masked words strongly activated language processing regions compared with nonwords – provided a baseline by which we could estimate the impact of priming personal goals compared with similar positively valenced trait attributes not motivationally relevant for the target participant.

The finding that the four priming conditions were associated with distinguishable patterns of activation reflecting both domain of goal and attainment status is a novel contribution of the present study. We were able to control experimentally for activation associated with positive trait attribute processing *per se* and look for activation patterns that represented neural signatures of personal goal pursuit as operationalized in RFT. The goal priming task is a subtle probabilistic manipulation that nonetheless provided a laboratory analog to the real-world situation in which a goal-relevant cue (either to “make something good happen” or to “keep something bad from happening”) appears in the individual’s social environment. In RFT itself, the two systems are postulated to involve distinct motivational inclinations, strategies, and distinct emotional reactions to success and failure feedback. As such, we would not have expected that the two “match” conditions (or the two “mismatch” conditions) would be associated with essentially identical activation patterns. Instead, we observed both commonalities and differences across the priming conditions that build upon and complement established network models of self-regulatory cognition. Accordingly, we consider the findings from the perspective of contributing to a growing “nomological net” linking constructs such as self-regulation with the cognitive neuroscience literature (De Vries et al., 2015).

### 3.1 Areas of activation common across promotion and prevention goal priming

We found activation across priming conditions in two regions well known as relevant to social cognition and self-regulation (Han, Bi, & Ybarra, 2016). There was one region (bilateral insula, BA 13), which showed significant activation in all four priming conditions on the TICA analyses. Both Heatherton and Langner et al. noted that the anterior insula was part of functional network involved in different aspects of self-control, and so this finding may represent a potential bridge between the two models emphasizing self-control and our own work which focuses more exclusively on goal pursuit *per se*. The insula is a region which has been implicated in many diverse affective and cognitive processes, including the integrations of multiple emotional states, response to informative feedback, and perhaps most relevant, self-agency (Contreras, Ceric, & Torrealba, 2007; Critchley, 2005; Farrer & Frith, 2002; Penfield & Faulk, 1955). It has also been demonstrated to functionally connect with the default mode network, possibly as part of a larger “environmental salience detection” network (Taylor, Seminowicz, & Davis, 2009) or as a component in a functional network that engages during self-evaluation (van der Cruijsen, Peters, & Crone, 2017). Additionally, the insula has been shown to be reactive during real-time fMRI feedback tasks (Li, Tong, Wang, Li, He, Guan, & Yan, 2016), suggesting at least the potential for engagement in voluntary aspects of goal pursuit as well as ongoing progress monitoring associated with efforts toward goal attainment.

For three of the four priming conditions (the exception being the ought/match condition), we observed activation in visual association cortex (BA 18/19) on either GLM or TICA analyses. Specifically, priming of personal goals was related to significant activity in cuneus, fusiform gyrus, and lingual gyrus. Those areas are emphasized by the cortical midline structures model, Heatherton and colleagues, and Langner and colleagues as critical to self-evaluation and self-concept, as well as other aspects of self-regulation. For example, activation in precuneus and cuneus has been linked with theory of mind (Schurz, Kronbichler, Weissengruber, Surtees, Samson, & Perner, 2015), guilt and empathy (Gifuni, Kendal, & Jollant, 2017), and cognitive dissonance (de Vries, Byrne, & Kehoe, 2015). Denny, Kober, Wager, and Ochsner (2012) reported functional associations between PFC and cuneus linked with self-judgment (compared with judgments about others). In addition, activation in the precuneus has been consistently associated with self-referential processing (Cavanna & Trimble, 2006; Feyers, Collette, D’Argembeau, Majerus, & Salmon, 2010), retrieval of autobiographical memories (Gardini et al., 2006; Loughead et al., 2010; McDermott, Szpunar, & Christ, 2009; Qin & Northoff, 2011), and self-projection (i.e., the ability to think about oneself in the future) (Buckner & Carroll, 2007). Likewise, fusiform gyrus has been implicated in social-cognitive processes including self-relevance (Moreira, Van Bavel, & Telzer, 2017) and self-other interaction (Saggar, Shelly, Lepage, Hoeft, & Reiss, 2014). The lingual gyrus has been found to be more active during autobiographical planning (i.e., planning for real-world personal goals) as opposed to visuospatial planning (Spreng et al., 2010) and is associated with intentionality (Golchert et al., 2017). It appears that personalized goal priming elicits activation which underlies the ability to contemplate, visualize, and plan for upcoming actions in service of both promotion and prevention goal pursuit.

### 3.2 Brain regions selectively engaged following promotion goal priming

In general, promotion goal priming engaged a larger set of brain regions than did prevention goal priming. Consistent with prior fMRI studies of promotion priming, both the ideal/match and ideal/mismatch priming conditions were associated with activation in and around left PFC (BA 9) including left middle frontal gyrus. Activation in response to both of the promotion goal priming conditions was significantly correlated with scores on the RFQ promotion success scale (but not the prevention success scale). These findings suggest that affective/motivational impetus in a promotion state is strategic approach, given the evidence that approach motivation and positive affectivity show a preference for left (vs. right) PFC (Coan & Allen, 2003; Harmon-Jones & Allen, 1998).

The ideal/mismatch priming condition was associated with the most widely distributed set of brain regions in the functional connectivity analyses. One potential explanation for this pattern involves the phenomenology of “mismatch” as operationalized in our personal goal assessment and fMRI paradigm. Specifically, the two “mismatch” priming conditions used positively valenced trait attributes which each participant had indicated previously they had not yet made progress toward attainment. While it is possible that for some individuals (for at least some of these priming words) such a goal-relevant cue might lead to a self-evaluation of failure, we believe that it is more conservative in this healthy young adult sample to interpret “mismatch” simply as signifying nonattainment. Therefore, the core similarity between the ideal/match and ideal/mismatch priming conditions was the invoking of personally salient promotion goals, but the key distinction would be that in the latter priming condition, the individual believed that they had yet to “make something good happen” with regard to those goals (Higgins, 1987, 1998). If that were the case, then the self-regulatory significance of the goal priming – once the cue is identified as a promotion goal – is that further strategizing and effort will be required. Assuming this phenomenological distinction corresponds to differences in brain activation, we may be able to learn a great deal about the neural correlates of personal goal pursuit by examining the match/mismatch distinction in greater depth in subsequent studies.

### 3.3 Brain regions selectively engaged following prevention goal priming

Although the two prevention priming conditions (ought/match and ought/mismatch) were associated with fewer regions of activation than the promotion priming conditions, our findings were consistent with previous studies and likewise support the discriminant validity of the distinction between promotion and prevention *per se*. When participants were primed with prevention attributes they felt they were close to attaining (ought match), activation was greater for control words in primarily in right PFC (BA 8 and 9, including right middle and superior frontal gyrus). Our findings suggest that priming with matching prevention goals (i.e., priming with goals one feels they have achieved in order to “keep bad things from happening”) results in greater right PFC activity, an activation pattern also consistent with the PFC asymmetry literature and with motivational strategies involving vigilance, caution, and even avoidance (Coan & Allen, 2003; Harmon-Jones & Allen, 1998). In contrast, ought/mismatch goal priming engaged bilateral caudate (unique to prevention priming) as well as visual association cortex (BA 18/19) regions shared across the four priming conditions. The former finding was unexpected but might be interpreted as consistent with previous studies that link caudate activation with goal-directed action, planning, the experience of guilt, and moral cognition (e.g., Mclatchie, Giner-Sorolla, & Derbyshire, 2016; Smith, Anand, Benattayallah, & Hodgson, 2015).

As noted above, the prevention goal priming conditions were associated with activation in fewer regions than promotion goal priming (despite their rough comparability with regard to percent variance accounted for in the analyses). What is clear is that promotion and prevention are distinguishable at both behavioral and neural levels, so until the findings can be replicated, we simply take them as indicative of a double-dissociation between the specific motivational neural correlates of the two hypothesized systems. Furthermore, the TICA analyses indicated found a significant control > ideal-match contrast in right middle and superior frontal cortex, as well as a significant control/ought-mismatch contrast in left middle frontal gyrus. Just as behavioral data suggest that at any given moment an individual can be in a promotion or prevention state but not both, so our activation data suggest the same functional distinction in the immediate aftermath of personal goal priming. In general, the findings replicated the left versus right prefrontal distinction previously observed for promotion versus prevention idiographic goal priming across the TICA and GLM analyses. In addition, however, we were able to identify overlap between the two kinds of motivational states which were consistent with the neuroscience of the self and with the three models we identified as especially likely to be relevant. The use of TICA allowed for identification of a somewhat broader functional network linked with promotion versus prevention specifically as well as with self-relevant goal priming *per se*. The TICA findings require replication, but they help to illuminate the neurocognitive processes underlying self-regulation as operationalized using RFT at a particular point in the ongoing cycle of personal goal pursuit (presentation of a motivationally significant goal cue). The investigators on whose models we drew in analyzing and interpreting our data have made clear that self-regulation is complex, multifaceted, and therefore likely to engage broad networks of brain regions detectable in both task-based and intrinsic functional connectivity analyses (seed-based as well as ICA-based).

Several limitations of the present work must be considered. First, we did not include a measure of the personal significance of the specific goals used as priming stimuli, which therefore does not allow us to rule out the possibility that some of the observed variances are a function of personal significance *per se* rather than domain (promotion vs. prevention) and attainment status (matching vs. mismatching). However, the idiographic goal assessment method that was used has been shown to be a reliable elicitor of personal goals and attributes that are chronically accessible and motivationally significant (Higgins & Bargh, 1987). Second, we did not manipulate chronic regulatory focus, and neither were participants selected on the basis of their RFQ scores or other measures of individual differences in the strength of the promotion versus prevention systems. Therefore, our analyses of those individual differences in regulatory focus are purely correlational in nature.

One more caveat is in order with regard to the present findings. As noted above, the priming manipulation we used represents one point in an ongoing cycle of self-regulation, and thus our findings should be interpreted as reflective of the individual’s initial response to personal goals (promotion and prevention, attained, or not attained). We would expect that different experimental paradigms would illuminate different points in that cycle, as well as functional associations with other well-established networks supporting goal-directed behavior (Satpute, Ochsner, & Badre, 2012) and emotion regulation (Braunstein, Gross, & Ochsner, 2017). Nonetheless, the substantial literature on the behavioral consequences of individual differences in promotion and prevention focus gives confidence that our findings may represent valid depictions of the neural correlates of each system. Even in light of these limitations, the present study clarifies the distinct neural correlates of the promotion and prevention systems and suggests that goal attainment status is an essential predictor of both behavioral and neural responses to personal goal priming.
